# Availability of a pediatric trauma center in a disaster surge decreases triage time of the pediatric surge population: a population kinetics model

**DOI:** 10.1186/1742-4682-8-38

**Published:** 2011-10-12

**Authors:** Erik R Barthel, James R Pierce, Catherine J Goodhue, Henri R Ford, Tracy C Grikscheit, Jeffrey S Upperman

**Affiliations:** 1 Children's Hospital Los Angeles, Division of Pediatric Surgery, 4650 Sunset Blvd, MS #100, Los Angeles, CA 90027, USA

## Abstract

**Background:**

The concept of disaster surge has arisen in recent years to describe the phenomenon of severely increased demands on healthcare systems resulting from catastrophic mass casualty events (MCEs) such as natural disasters and terrorist attacks. The major challenge in dealing with a disaster surge is the efficient triage and utilization of the healthcare resources appropriate to the magnitude and character of the affected population in terms of its demographics and the types of injuries that have been sustained.

**Results:**

In this paper a deterministic population kinetics model is used to predict the effect of the availability of a pediatric trauma center (PTC) upon the response to an arbitrary disaster surge as a function of the rates of pediatric patients' admission to adult and pediatric centers and the corresponding discharge rates of these centers. We find that adding a hypothetical pediatric trauma center to the response documented in an historical example (the Israeli Defense Forces field hospital that responded to the Haiti earthquake of 2010) would have allowed for a significant increase in the overall rate of admission of the pediatric surge cohort. This would have reduced the time to treatment in this example by approximately half. The time needed to completely treat all children affected by the disaster would have decreased by slightly more than a third, with the caveat that the PTC would have to have been approximately as fast as the adult center in discharging its patients. Lastly, if disaster death rates from other events reported in the literature are included in the model, availability of a PTC would result in a relative mortality risk reduction of 37%.

**Conclusions:**

Our model provides a mathematical justification for aggressive inclusion of PTCs in planning for disasters by public health agencies.

## Background

In the modern era, humanity has spread across and settled all habitable areas of the globe, thereby greatly increasing potential exposures to catastrophic events, whether natural or manmade, as demonstrated most recently by the 2010 Haiti earthquake [1] as well as the tragic earthquake, tsunami and nuclear disaster that devastated Japan in March, 2011 [2]. It is imperative that planning be undertaken to deal effectively with the vast number of injured survivors. These conditions can be described as a disaster surge, which can be thought of as an unusually high fluctuation over and above the normal background rate of patient utilization of medical services [3-12]. Multiple strategies have been proposed to maximize patient throughput and efficiency of resource utilization under surge conditions, and the overall consensus is that detailed planning for various disaster contingencies is the key to this process.

Because of the random, stochastic nature of disaster events, this planning can be greatly aided by simulation. A considerable amount of work has been done in modeling disaster surges and the response of health systems to them [13]. More generally, a patient population having to wait for medical triage and treatment can be thought of as a problem in queueing theory [14-17]. This field grew out of A. K. Erlang's pioneering approach to modeling demand for telephone service in the early 20^th ^century [18,19], and has been applied to a diverse range of problems including not only telecommunications, but airport and automobile traffic patterns, other service industries, and hospital and factory design [20-22]. If the length of the queue is long, then its behavior can often be approximated to that of a continuous variable, thereby simplifying the mathematics greatly. This approach results in what are referred to in the queueing theory literature as fluid models [23-25], and can be used for predicting the behavior of, for example, queues for service from a call-in center [26]. It has also been shown that if a system satisfies the Markov property, that is, if its future behavior depends only on its current state, then its behavior can be approximated deterministically by simple ordinary differential equations (ODE's) [27,28]. While more complicated stochastic methodologies such as Monte Carlo simulation have been successfully used in modeling the response to a patient surge [29,30], the simplicity of the ODE approach has motivated the use of kinetic or compartmental models for such problems [31]. In this method, the population evolves from an initial state to a number of subsequent states with each state change having a rate constant. This approach has also long been used in physics and chemistry to model reactions and series of reactions, as well as in population biology [32-34]. Here, we make use of this mathematically elementary and well-established approach to predict the behavior of pediatric and adult populations after a mass casualty event, with and without the availability of a facility specifically designed to treat children.

A significant proportion of disaster victims are children, who have unique physiology, patterns of injury, and psychosocial needs in such settings [35]. Studies have shown that the availability of a pediatric trauma center (PTC) would probably improve the overall response to a mass casualty incident, but the available data are sparse [36]. In the absence of more extensive data, in this paper we use a population kinetics approach to estimate the effect of the availability of a pediatric trauma center upon the rates of admission and discharge of a disaster surge population by extrapolating from historical data. We find that the initial rate of discharging patients from the PTC early in the surge is the dominant influence on the time needed to fill the hospital's maximum bed capacity as well as on the time needed to definitively treat and discharge all patients in the surge. On the other hand, the PTC admission rate and the rate of discharging patients once the PTC is full are the most important factors in determining the time needed to admit the entire surge. We then add historical mortality rates to our model and calculate the reduction in deaths that would be conferred by a PTC. We conclude that within the limits of our model, the availability of a PTC would greatly enhance the response to a disaster as measured by the total time needed to appropriately triage and treat the surge population.

## Methods

### I. Approach

Before describing the details of our model, we shall first solve a simpler problem that will provide its mathematical underpinnings. We begin by assuming that an unspecified disaster instantaneously produces an initial surge population. This scenario is a good approximation for a subset of mass casualty events (MCEs) that occur suddenly without appreciable buildup or exposure time, such as bombings, earthquakes, or airplane crashes. (The more general case, where there is a delay between the inciting event and the onset of the surge, is mathematically more complicated, requires more unknown parameters than the current scenario, and is developed for completeness in Appendix A.) This population, which we shall denote by *N_s_*(*t*), is defined at time zero to be *N_s_*(*t *= 0) = *N*_0_, and changes as it is admitted to a trauma center into a population *N_a_*(*t*) of admitted patients with rate *k_a_*, which in turn can become a population of *N_d_*(*t*) discharged patients with rate *k_d_*:

(1) $$N_{s}\overset{k_{a}}{\underset{}{\to }}N_{a}\overset{k_{d}}{\underset{}{\to }}N_{d}$$

Appropriate estimates for *k_a_*and *k_d_*will be discussed later when we apply our model to real-world historical data. We note that "discharge" would include mortality in this scheme, as no explicit provision is made for categories of discharge (discharged to home, discharged to a long term care facility, deceased, etc.). Equation 1 governs the behavior of the surge population as patients transition to being admitted and treated, and ultimately discharged; this behavior is described mathematically by a set of three coupled first-order differential equations:

(2) $$\frac{dN_{s}}{dt}=-k_{a}N_{s}$$

(3) $$\frac{dN_{a}}{dt}=k_{a}N_{s}-k_{d}N_{a}$$

(4) $$\frac{dN_{d}}{dt}=k_{d}N_{a}$$

To solve Eqs. 2-4, we require the boundary conditions:

(5) $$N_{s}\left(t=0\right)=N_{0}$$

(6) $$N_{s}\left(t\to \infty \right)=0$$

(7) $$N_{a}\left(t=0\right)=N_{a}\left(t\to \infty \right)=0$$

(8) $$N_{d}\left(t=0\right)=0$$

(9) $$N_{d}\left(t\to \infty \right)=N_{0}$$

Eqs. 5 and 6 state that the number of surge patients begins at *N*_0_, and decays to zero at long times since all patients are admitted and discharged. Eq. 7 reflects the fact that there are no patients admitted at time zero, and at long times all admitted patients have been discharged. Eqs. 8 and 9 therefore state that there are no discharged patients at time zero, while at long times the entire population has been discharged.

We can now solve the system of equations 2-4. Equation 2 can be solved by direct integration, and applying the boundary conditions 5 and 6 gives:

(10) $$N_{s}\left(t\right)=N_{0}e^{-k_{a}t}$$

Eq. 10 can be substituted into equation 3, yielding with some rearrangement:

(11) $$\frac{dN_{a}}{dt}+k_{d}N_{a}=k_{a}N_{0}e^{-k_{a}t}$$

Multiplying Eq. 11 by the integrating factor exp(*k_d_t*), integration and application of boundary condition (7) gives:

(12) $$N_{a}\left(t\right)=N_{0}\frac{k_{a}}{k_{d}-k_{a}}\left(e^{-k_{a}t}-e^{-k_{d}t}\right)$$

This can be substituted into Eq. 4, which after direct integration and application of boundary conditions (8) and (9) gives

(13) $$N_{d}\left(t\right)=N_{0}\left(\frac{k_{a}}{k_{d}-k_{a}}e^{-k_{d}t}-\frac{k_{d}}{k_{d}-k_{a}}e^{-k_{a}t}+1\right)$$

**Figure 1:**
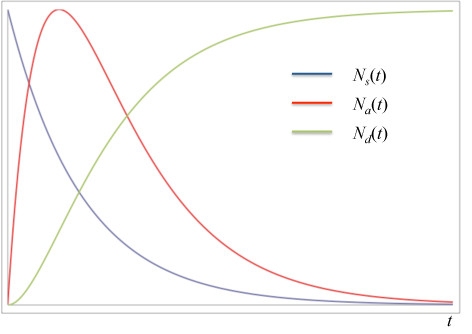
**Qualitative behavior of surge (blue), admitted (red), and discharged (green) populations with time as predicted by Equations 10, 12 and 13**. Curves are normalized for clarity.

Figure 1 shows a schematic of the behavior of the populations *N_s_*, *N_a_*, and *N_d_*as described by Equations 10, 12 and 13. No units are shown here for the sake of conceptual clarity; quantitative results are shown in the Results section. The surge population decays with typical single exponential behavior; the admitted population rises to a maximum and decays, and the discharged population exhibits an exponential rise.

### II. Maximum Capacity Model

At this point, we note that the model as currently formulated has a limitation in that no provision is made for the maximum capacity of the trauma center. In other words, the maximum value of *N_a_*(*t*) predicted by Eq. 12 is a function only of *N*_0_, *k_a_*and *k_d_*, with no dependence on the number of available beds in the center. To see this, *N_a_*(*t*) can be maximized by setting its derivative equal to zero and solving this expression for *t*, which gives

(14) tNamax=1kd-ka lnkdka

This value for *t *is inserted back into Eq. 12, giving

(15) $$N_{a}^{\max}=N_{0}\frac{k_{a}}{k_{d}-k_{a}}\left[{\left(\frac{k_{d}}{k_{a}}\right)}^{\frac{-k_{a}}{k_{d}-k_{a}}}-{\left(\frac{k_{d}}{k_{a}}\right)}^{\frac{-k_{d}}{k_{d}-k_{a}}}\right]$$

which is a function only of *N*_0_, *k_a_*and *k_d_*, and has no relation to any real-world hospital bed capacity.

**Figure 2:**
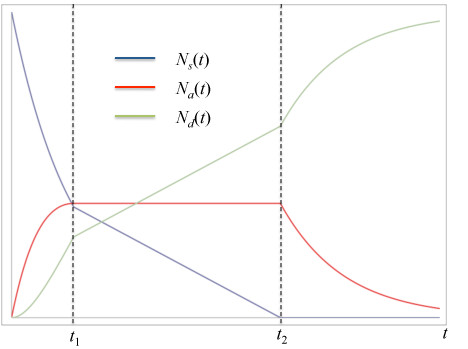
**Behavior of surge (blue), admitted (red), and discharged (green) populations with time in the maximum capacity model described by Equations 16-18**. Curves are not normalized or scaled.

This limitation can be overcome by modifying the model with some intuitive assumptions. First, we shall identify our trauma center's intrinsic maximum capacity as *N_a_*^max^, and assume that the surge population behaves just as we have described above until *N_a_*reaches *N_a_*^max^. This maximum census is not equal to the total number of beds in the trauma center, but rather its surge capacity over and above normal operations, or equivalently the fraction of its beds allotted in the center's planning for an MCE [37]. After *N_a_*^max ^is reached, we assume that the center will remain at maximum capacity until the surge is exhausted. That implies that the admission and discharge rates are equal during this period. Next, we assume that the admission rate will be somewhat lower after the trauma center is full compared with early times, as during this period many of its surge beds will be occupied with critically injured patients. Finally, we assume that once 100% of the surge has been admitted, the trauma center's discharge rate will return to that prior to maximum patient load. We will call this modified model the "maximum capacity model." To formulate this modification of the model mathematically, it is helpful to define two times *t*_1 _and *t*_2 _as illustrated in Figure 2. At *t*_1_, the trauma center has reached its maximum capacity and can only admit a patient if another is discharged, i.e., $t_{1}=t_{Na^{max}}$. This situation persists until *t*_2_, at which time the surge population has declined to zero and the trauma center can again discharge patients at the pre-MCE rate. In the language of queueing theory, *t*_2 _is the time at which the queue has vanished. The resulting population behavior is shown in Figure 2, where again, no units are shown for conceptual clarity; quantitative data are shown below in Results.

We are now ready to solve the necessary differential equations for the maximum capacity model. Overall, we have three separate regions in time with different behavior, defined by

(16) $$\begin{array}{ll} 0\leq t<t_{1}:\; & \frac{dN_{s}}{dt}=-k_{a}N_{s}\;\\ \;\;\;\;\;\;\; & \frac{dN_{a}}{dt}=k_{a}N_{s}-k_{d}N_{a}\;\\ \;\;\;\;\;\;\; & \frac{dN_{d}}{dt}=k_{d}N_{a}\;\\ & \; \end{array}$$

(17) $$\begin{array}{ll} t_{1}\leq t<t_{2}:\; & \frac{dN_{s}}{dt}=-k^{\prime }\;\\ \;\;\;\;\;\;\; & \frac{dN_{a}}{dt}=0\;\\ \;\;\;\;\;\;\; & \frac{dN_{d}}{dt}=k^{\prime }\;\\ & \; \end{array}$$

(18) $$\begin{array}{ll} t\geq t_{2}:\;\;\; & \frac{dN_{s}}{dt}=0\;\\ \;\;\;\;\;\;\; & \frac{dN_{a}}{dt}=-k_{d}N_{a}\;\\ \;\;\;\;\;\;\; & \frac{dN_{d}}{dt}=k_{d}N_{a}\;\\ & \; \end{array}$$

All variables and parameters have the same meanings as previously defined, except for a single new parameter *k' *that describes the admission and discharge rates during the period of time between *t*_1 _and *t*_2 _when the trauma center is operating at maximum capacity. We again note that *k' *will likely be less than either *k_a_*or *k_d_*, as both admissions and discharges will be slower once the trauma center is filled with critically injured surge patients.

The solution of Eq. 16 is identical to Equations 10, 12 and 13. However, at *t*_1 _the system's behavior changes to conform to Equation 17, giving

(19) $$\begin{array}{ll} t_{1}\leq t<t_{2}:\; & N_{s}=N_{s,t_{1}}-k^{\prime }\left(t-t_{1}\right)\;\\ \;\;\;\;\;\;\; & N_{a}=N_{a,t_{1}}\;\\ \;\;\;\;\;\;\; & N_{d}=N_{d,t_{1}}+k^{\prime }\left(t-t_{1}\right)\;\\ & \; \end{array}$$

where $N_{a,t_{1}}$= *N_a_*^max^. At *t*_2_, the entire surge population has been exhausted, and the system's behavior changes to that entailed by Eq. 18, the solution of which is

(20) $$\begin{array}{ll} t\geq t_{2}:\;\;\;\; & N_{s}=0\;\\ \;\;\;\;\;\;\; & N_{a}=N_{a,t_{2}}e^{-k_{d}\left(t-t_{2}\right)}\;\\ \;\;\;\;\;\;\; & N_{d}=N_{d,t_{2}}+N_{a,t_{2}}\left(1-e^{-k_{d}\left(t-t_{2}\right)}\right)\;\\ & \; \end{array}$$

We note that *t*_1 _has already been determined to be equal to$t_{Na^{max}}$ as defined in Eq. 14 and again $N_{a,t_{2}}=N_{a,t_{1}}$= *N_a_*^max^. We are now also in a position to determine *t*_2_, which is the time when *N_a_*= 0. Eq. 19 then gives

(21) $$t_{2}=t_{1}+\frac{N_{s,t_{1}}}{k^{\prime }}$$

### III. Maximum Capacity with Pediatric Trauma Center Model

Armed with Equations 16-18 we can now include the effect of an available pediatric trauma center in the maximum load model. We shall call what follows the "maximum capacity with pediatric trauma center model." We now assert that the initial *N*_0 _disaster victims are composed of *A*_0 _adults and *P*_0 _pediatric patients, viz:

(22) $$N_{0}=A_{0}+P_{0}$$

We also note that the total number of surge patients as a function of time is equal to the sum of the adult and pediatric subpopulations:

(23) $$N_{s}\left(t\right)=P_{s}\left(t\right)+A_{s}\left(t\right)$$

where *A_s_*and *P_s_*now indicate the adult and pediatric cohorts of the surge, respectively. We then assume that adult patients are only admitted to adult trauma centers, while pediatric patients may be triaged and admitted to either adult or pediatric trauma centers (PTCs); this assumption is similar to the approach taken by Perry and Whit in modeling call center capacity overloads [26], except that our case is asymmetric: adults are never triaged to PTCs in this model. These assumptions result in the following kinetic scheme:

(24) $$A_{s}\overset{k_{aa}}{\underset{}{\to }}A_{a}\overset{k_{ad}}{\underset{}{\to }}A_{d}$$

(25) $$P_{s}\overset{k_{paa}}{\underset{}{\to }}P_{aa}\overset{k_{pda}}{\underset{}{\to }}P_{d}$$

(26) $$P_{s}\overset{k_{pap}}{\underset{}{\to }}P_{ap}\overset{k_{pdp}}{\underset{}{\to }}P_{d}$$

where *k_aa_*and *k_ad_*represent the rates of adult admission to and discharge from an adult center, *k_paa_*and *k_pda_*the rates of pediatric admission to and discharge from the adult center, and *k_pap_*and *k_pdp_*the rates of pediatric admission to and discharge from the PTC. Similarly, *A_a_*(*t*) and *A_d_*(*t*) are the populations of admitted and discharged adults, while *P_aa_*(*t*) and *P_ap_*(*t*) are the pediatric populations admitted to adult and pediatric centers, respectively, and *P_d_*(*t*) represents the discharged pediatric population, irrespective of the center at which they were treated.

The differential equations entailed by Equation 24, boundary conditions, and their solution are identical to Equations 1-4 except for subscripts:

(27) $$A_{s}\left(t\right)=A_{0}e^{-k_{aa}t}$$

(28) $$A_{a}\left(t\right)=A_{0}\frac{k_{aa}}{k_{ad}-k_{aa}}\left(e^{-k_{aa}t}-e^{-k_{ad}t}\right)$$

(29) $$A_{d}\left(t\right)=A_{0}\left(\frac{k_{aa}}{k_{ad}-k_{aa}}e^{-k_{ad}t}-\frac{k_{ad}}{k_{ad}-k_{aa}}e^{-k_{aa}t}+1\right)$$

On the other hand, the coupled differential equations resulting from Equations 25-26 are slightly different:

(30) $$\frac{dP_{s}}{dt}=-\left(k_{paa}+k_{pap}\right)P_{s}$$

(31) $$\frac{dP_{aa}}{dt}=k_{paa}P_{s}-k_{pda}P_{aa}$$

(32) $$\frac{dP_{ap}}{dt}=k_{pap}P_{s}-k_{pdp}P_{ap}$$

(33) $$\frac{dP_{d}}{dt}=k_{pda}P_{aa}+k_{pdp}P_{ap}$$

The differences arise from the fact that there are potentially different rates of admission of pediatric patients to, and discharge of these patients from, the adult and pediatric trauma centers in the model. If the admission and discharge rates are equal, Equations 31-32 collapse into a single equation that is analogous to (1) and (24). The boundary conditions on (30) and (33) are the same as (5-6) and (8-9); those for (31) and (32) are identical to (7). At this point it is helpful to define:

(34) $$k\equiv k_{paa}+k_{pap}$$

Eq. 26 essentially defines an effective or total admission rate constant for pediatric patients in the model. The solution to (30-33), though slightly more complicated, is obtained via the same algorithm that led to (10-13) and is as follows:

(35) $$P_{s}\left(t\right)=P_{0}e^{-kt}$$

(36) $$P_{aa}\left(t\right)=P_{0}\frac{k_{paa}}{k_{pda}-k}\left(e^{-kt}-e^{-k_{pda}t}\right)$$

(37) $$P_{ap}\left(t\right)=P_{0}\frac{k_{pap}}{k_{pdp}-k}\left(e^{-kt}-e^{-k_{pdp}t}\right)$$

(38) $$\begin{array}{c} P_{d}\left(t\right)=P_{0}\left[\frac{k_{paa}}{k_{pda}-k}e^{-k_{pda}t}+\frac{k_{pap}}{k_{pdp}-k}e^{-k_{pdp}t}\right)\\ \;\;\;\;\;\left(+\left(\frac{k_{pda}k_{paa}}{k\left(k-k_{pda}\right)}+\frac{k_{pdp}k_{pap}}{k\left(k-k_{pdp}\right)}\right)e^{-kt}+1\right] \end{array}$$

**Figure 3:**
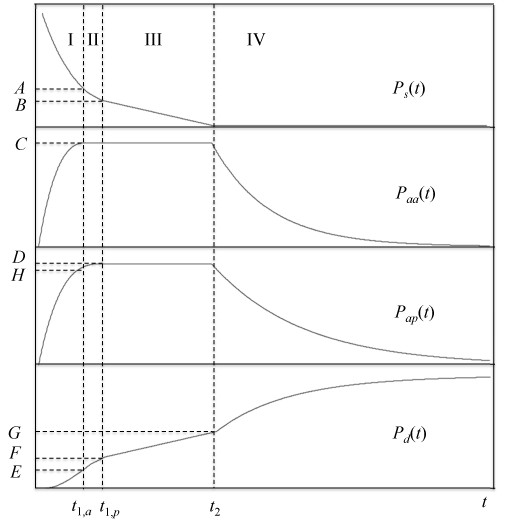
**Behavior of pediatric cohort of the surge (top panel), admissions to adult center (second panel), admissions to pediatric center (third panel), and discharged patients (fourth panel) with time as predicted by the maximum capacity with PTC model, Eqs. 40-50**. Uppercase letters indicate boundary conditions for the appropriate differential equations; see text for details.

Equations 35-38 describe the behavior of the pediatric cohort of the surge prior to the maximum load times for the adult and pediatric trauma centers. The behavior will change to one similar to Eq. 19 after these maxima are reached. However, there is no longer a single time for the maximum load, but rather separate ones for the adult and pediatric centers. Moreover, the behavior between the maximum load for the faster facility and that of the slower one requires a separate set of differential equations to be solved. To show this, Figure 3 displays the qualitative behavior of the model we are about to derive, with the necessary boundary conditions and regions where each set of differential equations holds (I, II, III and IV) noted on the figure. The time for maximum load for the center that admits and discharges patients more rapidly is analogous to Eq. 14. For the purposes of developing the model, we shall assume that the adult center is faster, but the derivation proceeds identically if the opposite assumption is made, except for the subscripts on the parameters; this issue is discussed further in additional files 1 and 2. We shall also omit the constant prefactor *P*_0 _from all the equations that follow, since it can be added back in after the derivation is complete with no loss of generality. Therefore:

(39) $$t_{1,a}\equiv t_{paa}^{max}=\frac{1}{k_{pda}-k}ln\left(\frac{k_{pda}}{k}\right)$$

The equations governing the system's behavior in region II of Figure 3 are

(40) $$\begin{array}{ll} t_{1,a}\leq t<t_{1,p}:\; & \frac{dP_{s}}{dt}=-{k_{a}}^{\prime }-k_{pap}P_{s}\;\\ \;\;\;\;\;\;\;\; & \frac{dP_{aa}}{dt}=0\;\\ \;\;\;\;\;\;\;\; & \frac{dP_{ap}}{dt}=k_{pap}P_{s}-k_{pdp}P_{ap}\;\\ \;\;\;\;\;\;\;\; & \frac{dP_{s}}{dt}={k_{a}}^{\prime }+k_{pdp}P_{ap}\;\\ & \; \end{array}$$

The solution to these equations is

(41) $$\begin{array}{c} t_{1,a}\leq t<t_{1,p}:\\ \;\;P_{s}=-\frac{{k_{a}}^{\prime }}{k_{pap}}+\alpha e^{-k_{pap}\left(t-t_{1,a}\right)}\\ \;\;P_{aa}=C\\ \;\;P_{ap}=\frac{{k_{a}}^{\prime }}{k_{pdp}}\left(e^{-k_{pdp}\left(t-t_{1,a}\right)}-1\right)\\ \;\;\;\;+\gamma \left(e^{-k_{pap}\left(t-t_{1,a}\right)}-e^{-k_{pdp}\left(t-t_{1,a}\right)}\right)+He^{-k_{pdp}\left(t-t_{1,a}\right)}\\ \;\;P_{d}=\left(H-\gamma +\frac{{k_{a}}^{\prime }}{k_{pdp}}\right)\left(1-e^{-k_{pdp}\left(t-t_{1,a}\right)}\right)\\ \;\;\;\;+\gamma \frac{k_{pdp}}{k_{pap}}\left(1-e^{-k_{pap}\left(t-t_{1,a}\right)}\right)+E \end{array}$$

where the constants are given by

(42) $$\begin{array}{c} \alpha =A+\frac{{k_{a}}^{\prime }}{k_{pap}}\\ \gamma =\frac{Ak_{pap}+{k_{a}}^{\prime }}{k_{pdp}-k_{pap}}\\ A=e^{-kt_{1,a}}\\ C=\frac{k_{paa}}{k_{pda}-k}\left(A-e^{-k_{pda}t_{1,a}}\right)\\ E=\frac{k_{paa}}{k_{pda}-k}e^{-k_{pda}t_{1,a}}+\frac{k_{pap}}{k_{pdp}-k}e^{-k_{pdp}t_{1,a}}\\ \;\;\;\;+\left(\frac{k_{pda}k_{paa}}{k\left(k-k_{pda}\right)}+\frac{k_{pdp}k_{pap}}{k\left(k-k_{pdp}\right)}\right)A+1\\ H=\frac{k_{pap}}{k_{pdp}-k}\left(A-e^{-k_{pdp}t_{1,a}}\right) \end{array}$$

The time for the maximum load *D *on the slower center (the pediatric trauma center in this derivation) can be found by maximizing the expression for *P_ap_*in (41). Thus the expressions for *t*_1,*p *_and *D *are:

(43) $$\begin{array}{c} t_{1,p}=\frac{1}{k_{pap}-k_{pdp}}ln\left(\frac{\alpha k_{pap}}{\beta k_{pdp}}-\frac{\gamma }{\beta }\right)\\ D=P_{ap}\left(t_{1,p}\right)=\frac{{k_{a}}^{\prime }}{k_{pdp}}\left(e^{-k_{pdp}\left(t_{1,p}-t_{1,a}\right)}-1\right)\\ \;\;\;\;+\gamma \left(e^{-k_{pap}\left(t_{1,p}-t_{1,a}\right)}-e^{-k_{pdp}\left(t_{1,p}-t_{1,a}\right)}\right)\\ \;\;\;\;+He^{-k_{pdp}\left(t_{1,p}-t_{1,a}\right)} \end{array}$$

with β given by:

(44) $$\beta =H-\gamma +\frac{{k_{a}}^{\prime }}{k_{pdp}}$$

*B *and *F *are also obtained by substituting *t*_1,*p *_into the appropriate expressions in (41):

(45) $$\begin{array}{c} B=-\frac{{k_{a}}^{\prime }}{k_{pap}}+\alpha e^{-k_{pap}\left(t_{1,p}-t_{1,a}\right)}\\ F=\left(H-\gamma +\frac{{k_{a}}^{\prime }}{k_{pdp}}\right)\left(1-e^{-k_{pdp}\left(t_{1,p}-t_{1,a}\right)}\right)\\ \;\;\;\;+\gamma \frac{k_{pdp}}{k_{pap}}\left(1-e^{-k_{pap}\left(t_{1,p}-t_{1,a}\right)}\right)+E \end{array}$$

After *t*_1, *p*_, the slower pediatric center is also at its maximum capacity, and both centers can admit a patient only if another is discharged. The kinetics then becomes zeroth order similarly to Eqs. 17 and 19, so in region III of Figure 3, the governing equations are

(46) $$\begin{array}{ll} t_{1,p}\leq t<t_{2}:\;\; & \frac{dP_{s}}{dt}=-\kappa \;\\ \;\;\;\;\;\;\;\; & \frac{dP_{aa}}{dt}=\frac{dP_{ap}}{dt}=0\;\\ \;\;\;\;\;\;\;\; & \frac{dP_{d}}{dt}=\kappa \;\\ & \; \end{array}$$

where κ = *k_a_' *+ *k_p_'*, the sum of the discharge rates of the adult and pediatric centers during the period of time while they are at maximum capacity (region III). The solution to (46) is

(47) $$\begin{array}{ll} t_{1,p}\leq t<t_{2}:\; & P_{s}=B-\kappa \left(t-t_{1,p}\right)\;\\ \;\;\;\;\;\;\; & P_{aa}=C\;\\ \;\;\;\;\;\;\; & P_{ap}=D\;\\ \;\;\;\;\;\;\; & P_{d}=F+\kappa \left(t-t_{1,p}\right)\;\\ & \; \end{array}$$

The value for *t*_2_, the time when the surge is exhausted, is just the *t*-intercept of the line describing *P_s_*in region III. This can also be used to find *G*:

(48) $$\begin{array}{c} t_{2}=t_{1,p}+\frac{B}{\kappa }\\ G=F+\kappa \left(t_{2}-t_{1,p}\right)=F+B \end{array}$$

Because the surge cohort has vanished at *t*_2_, there is no more external load upon either trauma center, so we again make the assumption that each center can resume discharging patients at the pre-MCE rate as in Eq. 18. This is only a simplifying assumption, as arbitrary rates could be assumed with no effect on the derivation except for subscripts. The differential equations for region IV are then

(49) $$\begin{array}{ll} t>t_{2}:\; & \frac{dP_{s}}{dt}=P_{s}=0\;\\ \;\;\;\; & \frac{dP_{aa}}{dt}=-k_{pda}P_{aa}\;\\ \;\;\;\; & \frac{dP_{ap}}{dt}=-k_{pdp}P_{ap}\;\\ \;\;\;\; & \frac{dP_{d}}{dt}=k_{pda}P_{aa}+k_{pdp}P_{ap}\;\\ & \; \end{array}$$

All boundary conditions are known and illustrated in Figure 3, including that at long times *P_d_*must be unity. Therefore the solution to (49) is:

(50) $$\begin{array}{ll} t>t_{2}:\; & P_{s}=0\;\\ \;\;\;\; & P_{aa}=Ce^{-k_{pda}\left(t-t_{2}\right)}\;\\ \;\;\;\; & P_{ap}=De^{-k_{pdp}\left(t-t_{2}\right)}\;\\ \;\;\;\; & P_{d}=1-Ce^{-k_{pda}\left(t-t_{2}\right)}-De^{-k_{pdp}\left(t-t_{2}\right)}\;\\ & \; \end{array}$$

## Results

### I. Application of the maximum capacity model to an historical example

Equations 40-50 now allow us to examine the behavior of the pediatric surge population *P*_0 _under a variety of conditions. We begin by identifying the appropriate parameters in the simpler maximum capacity model that define real-world timescales. We then proceed to work through an example of applying the model by considering literature admission and discharge data from an historical disaster surge. We fit the equations to these data, and then include the full maximum capacity with pediatric trauma center model to extrapolate the effect a pediatric trauma center would have had on the time necessary to treat the patients.

There are several potentially observable parameters in the models presented here. The rates of admission and discharge in the initial and maximum capacity regimes are certainly observable in principle, but they are rarely reported as such. Also, the maximum surge capacities *N_a_*^max^, *C *and *D *are available to disaster planners, but not usually reported directly. Rather, what is often available are the times of maximum load (*t*_1 _in the maximum capacity model, *t*_1,*a *_and *t*_1,*p *_in the maximum capacity model with pediatric trauma center available) and the time at which the surge population has been completely dispositioned. The latter time does not correspond to *t*_2_, since the trauma centers are still full to capacity at this point. Rather, this is the time at which, in region IV of Figure 3, the discharged population has increased to very nearly unity. We note that it cannot be defined as the time that exactly 100% of the surge has been discharged, since the exponentials governing the behavior of the populations do not reach this value until infinity. Rather, we can define a time at which some specified fraction of discharges has been reached: we shall choose 99% and call this time *t*_99_. From Equation 20, it follows that in the maximum capacity model *t*_99 _is given by

(51) t99=t2-1kd ln1-0.99-Nd,t2Na,t2

However, in the maximum capacity with PTC model, Equation 50 is transcendental so *t*_99 _cannot be solved for in closed form, but it can be found numerically. We note that our choice of the parameters *t*_1 _and *t*_99 _was motivated in large part by the availability of such data in the literature, but also by the importance of *t*_1 _as a defining timescale of the behavior of populations in the model. On the other hand, we include *t*_2 _primarily as a natural timescale of the model itself (where the surge or queue length vanishes and the system's deterministic behavior changes again) rather than as a descriptor of available historical data, and we examine the effect of varying it in the sensitivity analysis. Finally, the effect of including the explicit contribution of death rates for each population is derived in Appendix B.

The historical example we shall use in demonstrating the implementation of the model is that of an Israeli Defense Forces mobile field hospital that responded to the 2010 Haiti earthquake. In that case, 1111 patients were treated over a course of 10 days, and the hospital's maximum capacity of 60 to 72 beds was reached prior to 2 days of operation [1]. We chose this example because the disaster itself was sudden as required by our assumptions, and because of the quality of the data available with which to fit our model in comparison to other historical MCEs. We will begin by fitting the maximum capacity model (without a pediatric trauma center available) to these data. This amounts to solving a system of equations consisting of (13), (14) and (21) for *k_a_*, *k_d_*, and *k' *with the historical data of *t*_1 _= 2 days, *t*_99 _= 10 days. Since we have three equations with two unknowns, the system is not uniquely determined and actually has two solutions, one for the case of *k_a_*>*k_d_*and another for *k*_a _<*k*_d_. To overcome this we must impose a constraint for *t*_2_: for this case we shall arbitrarily assume that it took approximately the same time to discharge all admitted patients once the surge was exhausted as it did for the hospital to reach maximum capacity, that is, *t*_2 _= 8. In other words, we are requiring in this example that

(52) $$t_{99}-t_{2}=t_{1}$$

With this constraint, the model can be numerically solved uniquely given the historical data. This assumption could be eliminated if real historical data were available for *t*_2_, and we examine the effect of varying this constraint in the sensitivity analysis. The results given the observed data and the constraint (52) are *k_a_*= 0.158 ± 0.066 day^-1^, *k_d_*= 1.151 ± 0.377 day^-1^, *k' *= 0.122 ± 0.014 day^-1^; the uncertainties are one standard deviation. The model was fit using the Frontline Systems (Incline Village, NV, USA) Solver add-in for Microsoft Excel 2008 for Macintosh. To obtain estimates of parameter uncertainties, we assumed unit variance for the input data *t*_1_, *t*_2_, and *t*_99_. We then fit the sums of squared errors as polynomial functions of the parameters *k_a_*, *k_d_*, and *k'*, obtained their derivatives, and approximated the variances of the parameters as twice the inverse of the second derivative of the error with respect to each (neglecting covariances), as in [38]. We can now use these results as our baseline and proceed to add a hypothetical pediatric trauma center to this example as part of our sensitivity analysis.

### II. Sensitivity analysis

#### A. Approach

In general, the output of a mathematical model depends upon the model methodology and the input parameters. Accordingly, the sensitivity of the output to the uncertainties in the parameters fit to experimental data can be assessed in a formal sensitivity analysis. For kinetic models of this type, much work has been published in the physics and chemistry literature on methods to perform this analysis [39-42], but in this section we follow Atherton et al.'s approach [41]. In this section of the paper, we apply this methodology to fits obtained with the maximum capacity model in the previous section. In addition, though literature values are not available for some of the parameters in the more complicated maximum capacity with PTC model, we shall also make predictions about the effects of the availability of a pediatric trauma center on triage and discharge times if some reasonable assumptions are made about these parameters. Lastly we shall address mortality of the surge population using a modification of the model that includes explicit death rates of each population and is fully derived in Appendix B.

In the maximum capacity model, there are three parameters, *k_a_*, *k_d_*, and *k'*, and three outputs, *t*_1_, *t*_2 _and *t*_99_. In general, if covariances are neglected, the variance or squared standard deviation of the *i*th output *X_i_*, in terms of the parameters *p_j_*of a model, is:

(53) $$\sigma_{X_{i}}^{2}=\underset{j}{\sum }{\left(\frac{\partial X_{i}}{\partial p_{j}}\right)}^{2}\sigma_{p_{j}}^{2}$$

In our case, the sum index *j *runs from one to three for the three parameters for each output variable. Therefore, there are nine elements of the relevant sensitivity matrix **S**, with the matrix elements given by

(54) $$S_{ij}=\frac{\partial X_{i}}{\partial p_{j}}$$

We can then define an output variance matrix **V **with the matrix elements

(55) $$V_{ij}=S_{ij}^{2}\sigma_{p_{j}}^{2}$$

Again following Atherton et al., the effects of parameter uncertainties on the *i*th output variable are then ranked in order of their magnitude.

To accomplish this, we require the nine partial derivatives implied by Equation 54, which are shown below:

(56) $$\frac{\partial t_{1}}{\partial k_{a}}=\frac{1}{k_{d}-k_{a}}\left(t_{1}-\frac{1}{k_{a}}\right)$$

(57) $$\frac{\partial t_{1}}{\partial k_{d}}=\frac{1}{k_{d}-k_{a}}\left(\frac{1}{k_{d}}-t_{1}\right)$$

(58) $$\frac{\partial t_{1}}{\partial k^{\prime }}=0$$

(59) $$\frac{\partial t_{2}}{\partial k_{a}}=\left(1-\frac{k_{a}}{k^{\prime }}e^{-k_{a}t_{1}}\right)\frac{\partial t_{1}}{\partial k_{a}}-\frac{t_{1}}{k^{\prime }}e^{-k_{a}t_{1}}$$

(60) $$\frac{\partial t_{2}}{\partial k_{d}}=\left(1-\frac{k_{a}}{k^{\prime }}e^{-k_{a}t_{1}}\right)\frac{\partial t_{1}}{\partial k_{d}}$$

(61) $$\frac{\partial t_{2}}{\partial k^{\prime }}=-\frac{1}{{k^{\prime }}^{2}}e^{-k_{a}t_{1}}$$

(62) $$\frac{\partial t_{99}}{\partial k_{a}}=\frac{\partial t_{2}}{\partial k_{a}}-\frac{1}{k_{d}}\left[\frac{\frac{\partial N_{d,t_{2}}}{\partial k_{a}}+\left(\frac{0.99-N_{d,t_{2}}}{N_{a,t_{2}}}\right)\frac{\partial N_{a,t_{2}}}{\partial k_{a}}}{N_{a,t_{2}}-\left(0.99-N_{d,t_{2}}\right)}\right]$$

(63) $$\begin{array}{c} \frac{\partial t_{99}}{\partial k_{d}}=\frac{1}{k_{d}^{2}}ln\left(1-\frac{0.99-N_{d,t_{2}}}{N_{a,t_{2}}}\right)+\frac{\partial t_{2}}{\partial k_{d}}\\ \;\;\;\;-\frac{1}{k_{d}}\left[\frac{\frac{\partial N_{d,t_{2}}}{\partial k_{d}}+\left(\frac{0.99-N_{d,t_{2}}}{N_{a,t_{2}}}\right)\frac{\partial N_{a,t_{2}}}{\partial k_{d}}}{N_{a,t_{2}}-\left(0.99-N_{d,t_{2}}\right)}\right] \end{array}$$

(64) $$\frac{\partial t_{99}}{\partial k^{\prime }}=\frac{\partial t_{2}}{\partial k^{\prime }}$$

with the first partial derivative in the numerator of the bracketed expression in Equation 62 given by

(65) $$\frac{\partial N_{d,t_{2}}}{\partial k_{a}}=\frac{\partial N_{d,t_{1}}}{\partial k_{a}}+k^{\prime }\left(\frac{\partial t_{2}}{\partial k_{a}}-\frac{\partial t_{1}}{\partial k_{a}}\right)$$

where the first term in Equation 65 is

(66) $$\begin{array}{c} \frac{\partial N_{d,t_{1}}}{\partial k_{a}}=\left(e^{-k_{d}t_{1}}-e^{-k_{a}t_{1}}\right)\left(\frac{k_{d}}{{\left(k_{d}-k_{a}\right)}^{2}}-\frac{k_{a}k_{d}}{k_{d}-k_{a}}\frac{\partial t_{1}}{\partial k_{a}}\right)\\ \;\;\;+t_{1}\frac{k_{d}}{k_{d}-k_{a}}e^{-k_{a}t_{1}} \end{array}$$

The other partial derivative in the bracketed expression in Equation 62 is

(67) $$\begin{array}{c} \frac{\partial N_{a,t_{2}}}{\partial k_{a}}=\left(\frac{k_{a}}{k_{d}-k_{a}}\right)\left[\left(k_{d}e^{-k_{d}t_{1}}-k_{a}e^{-k_{a}t_{1}}\right)\frac{\partial t_{1}}{\partial k_{a}}-t_{1}e^{-k_{a}t_{1}}\right]\\ \;\;\;+\frac{k_{d}}{{\left(k_{d}-k_{a}\right)}^{2}}\left(e^{-k_{a}t_{1}}-e^{-k_{d}t_{1}}\right) \end{array}$$

Similarly, the required expressions to evaluate Equation 63 are

(68) $$\frac{\partial N_{d,t_{2}}}{\partial k_{d}}=\frac{\partial N_{d,t_{1}}}{\partial k_{d}}+k^{\prime }\left(\frac{\partial t_{2}}{\partial k_{d}}-\frac{\partial t_{1}}{\partial k_{d}}\right)$$

(69) $$\begin{array}{c} \frac{\partial N_{d,t_{1}}}{\partial k_{d}}=\left(e^{-k_{a}t_{1}}-e^{-k_{d}t_{1}}\right)\left(\frac{k_{a}}{{\left(k_{d}-k_{a}\right)}^{2}}+\frac{k_{a}k_{d}}{k_{d}-k_{a}}\frac{\partial t_{1}}{\partial k_{d}}\right)\\ \;\;\;-t_{1}\frac{k_{a}}{k_{d}-k_{a}}e^{-k_{d}t_{1}} \end{array}$$

(70) $$\begin{array}{c} \frac{\partial N_{a,t_{2}}}{\partial k_{d}}=\left(\frac{k_{a}}{k_{d}-k_{a}}\right)\left[\left(k_{d}e^{-k_{d}t_{1}}-k_{a}e^{-k_{a}t_{1}}\right)\frac{\partial t_{1}}{\partial k_{d}}+t_{1}e^{-k_{d}t_{1}}\right]\\ \;\;\;-\frac{k_{a}}{{\left(k_{d}-k_{a}\right)}^{2}}\left(e^{-k_{a}t_{1}}-e^{-k_{d}t_{1}}\right) \end{array}$$

Substituting the appropriate values for the rate constants and outputs from the maximum capacity model gives

(71) $$S=\left(\begin{array}{ccc} -4.36 & -1.14 & 0\\ -12.23 & -0.06 & -49.37\\ -7.87 & -2.40 & -49.37 \end{array}\right)$$

where the first row gives the derivatives for *t*_1_, the second for *t*_2_, and the third for *t*_99_, and the columns correspond to differentiation with respect to *k_a_*, *k_d_*and *k*', respectively. After squaring each element and multiplying each column by the variance of the appropriate parameter, we finally obtain

(72) $$V=\left(\begin{array}{ccc} 0.082 & 0.184 & 0\\ 0.649 & 0.0005 & 0.500\\ 0.268 & 0.815 & 0.500 \end{array}\right)$$

#### B. Effect of varying the constraint *t_99 _- t_2_*

**Figure 4:**
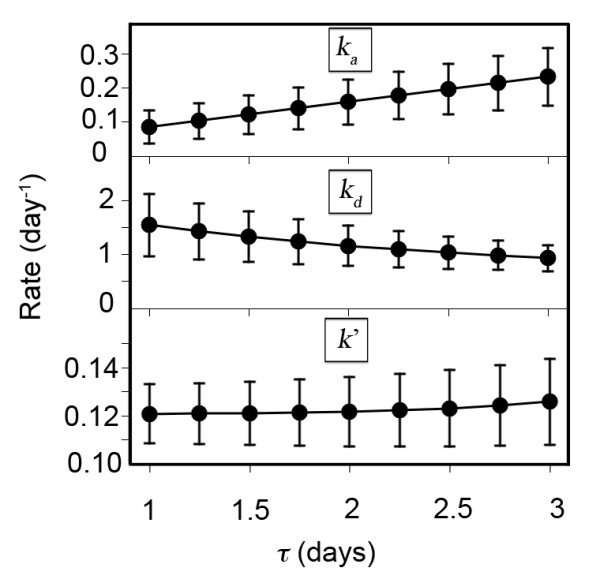
**Effect of changing the constraint on τ = t2-t1 on the fitted values of the rates for the maximum capacity model**. Error bars represent one standard deviation; see text for details.

As noted in our introduction of Equation 52, in order to obtain a unique solution for the fit of the maximum capacity model to the data available from Reference [1], we had to impose a constraint on the difference between the time at which 99 percent of the patients had been discharged and the time at which the patient surge was exhausted. We arbitrarily assumed that this difference would be equal to the time needed to evolve from time zero to steady state, *t*_1 _in the maximum capacity model. To determine the effect of relaxing this constraint, we varied this difference by ± 50%, i.e., we defined

(73) $$\tau =t_{99}-t_{2}$$

we varied τ from 1 to 3 days and re-fit the data, bracketing our initial constraint of 2 days. The net effect of this approach is to vary *t_2_*, because *t_99_*is fixed at 10 days by the historical data. The results of this calculation are shown in Figure 4, which depicts the three rate constants *k_a_*, *k_d_*, and *k*' as a function of τ. Since *t*_1 _is also constant and fixed by the ratio of *k_d_*to *k_a_*, as *k_d_*decreases with increasing τ, *k_a_*must increase accordingly. Because *t_99_*is fixed, and by Equation 20 the behavior of the maximum capacity model in region III is governed by *k_d_*, a smaller *k_d_*results in a larger τ and a shorter time spent in the steady state regime of region II. Therefore, the steady state discharge rate *k*' must therefore increase with increasing τ, which is indeed the case.

#### C. Availability of a Pediatric Trauma Center speeds admission of the pediatric cohort

We are now in a position to include the hypothetical effect predicted by the maximum capacity with PTC model of the availability of a pediatric trauma center upon the flow of pediatric patients in this historical example. The approach we take is to vary the three parameters *k_pap_*, *k_pdp_*, and *k_p_*' from much less than the corresponding adult center parameters *k_paa_*, *k_pda_*, and *k_a_*' smoothly up to the latter values fitted from our historical example. We then determine the effect on observable quantities *t*_2 _and *t*_99 _from the model, the times needed to completely admit and discharge the surge population, respectively. For this paper, we did not independently vary the three parameters from zero to the fitted adult values. Rather, we first chose to look at a subset of the parameter space, that in which the pediatric parameters are uniformly scaled by a single factor, ranging from much less than one up to nearly one, multiplied by the corresponding adult parameters. Our rationale in this approach was that without historical data for the ratios of the pediatric admission and discharge rates to one another, it was reasonable to fix them to the proportions between those of the adult center, for which, in contrast, we were able to fit available data. At this point, we also recall that in the derivation of the model, we assumed that the steady state discharge rates *k_p_*' and *k_a_*' were less than their corresponding discharge rates prior to achieving maximum capacity, *k_pdp_*and *k_pda_*, which restricts the parameter space available to explore, though this had no effect on the analysis that follows.

**Figure 5:**
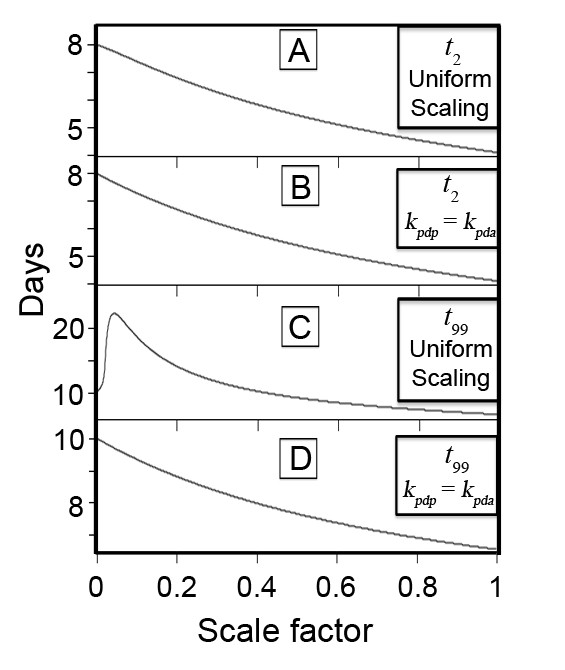
**Time needed to admit (t2, panels A and B) and definitively treat to discharge (t99, panels C and D) the pediatric surge population for two subsets of the input parameter space**. When the pediatric center's admission and discharge rate parameters are uniformly scaled up from zero to the values for the adult center, t2 decreases monotonically but t99 is initially prolonged (A, C). If the PTC discharge rate is set equal to that of the adult center for the pediatric surge patients while the admission and steady-state discharge rates are scaled up from zero to the values for the adult center, t2 (panel B) behaves similarly as in A, but t99 decreases uniformly (D). See text for details.

Figure 5 shows the effect on *t*_2 _and *t*_99 _of varying the pediatric parameters from a factor of 10^-3 ^times the fitted adult parameters up to a factor of 0.999, and some clear behavior emerges. It can be seen that despite the monotonic decrease in *t*_2 _as the pediatric center's effect is scaled up from near zero to approaching that of the adult center (Figure 5A), there is an initial increase in *t*_99 _that peaks at a scale factor of approximately 0.04, and this only falls below the baseline value of 10 days when the pediatric parameters are scaled by 0.4 or greater (Figure 5C). We hypothesized that this effect arose largely from trapping of patients in the pediatric center when it was unable to discharge them at a sufficient rate. To test this, we investigated a second case where the pediatric discharge rate was fixed at the adult rate for all values of *k_paa_*and *k_a_*', and the latter two were scaled as in the first case. As shown in Figure 5B and 5D, if *k_pdp_*is set equal to *k_pda_*the prolongation of *t*_99 _is eliminated and both *t*_2 _and *t*_99 _decrease as *k_paa_*and *k_a_*' are scaled from near zero to the adult values.

The initial increase of *t*_99 _for small uniform scale factors can be explained in greater detail by examining the behavior of the population of discharged patients in the maximum capacity with PTC model at long times when this factor is small. In this case, we can write:

(74) $$k_{pdp}=\varepsilon k_{pda}$$

where *ε *< < 1. The population of discharged patients, the final expression in Equation 50, can then be approximated at long times by

(75) $$P_{d}\approx 1-De^{-k_{pdp}\left(t-t_{2}\right)}$$

since the *C *term decays much faster than the *D *term. It is then easy to show that

(76) $$t_{99}=t_{2}-\frac{1}{\varepsilon k_{pda}}\text{ln}\left(\frac{0.01}{D}\right)$$

The derivative of *t*_99 _with respect to *ε *is then

(77) $$\frac{\partial t_{99}}{\partial \varepsilon }=\frac{\partial t_{2}}{\partial \varepsilon }+\frac{1}{\varepsilon^{2}k_{pda}}\text{ln}\left(\frac{0.01}{D}\right)+\frac{1}{\varepsilon k_{pda}}\frac{\partial lnD}{\partial \varepsilon }$$

We must show that the right hand side of Equation 77 is positive for small but finite positive scale factor ε. Although *D *is a nonlinear function of *k_pdp_*(cf. Equation 43) and therefore of *ε *in this approximation, its behavior is constrained by physical considerations that allow for a simple justification of this hypothesis. First, since *D *is the proportion of inpatients admitted to the pediatric trauma center after steady state has been achieved in Region III, it can never be negative, and it must necessarily be identically zero if the rate of admission to the PTC is also zero, or equivalently, if *ε *vanishes. Secondly, for very small but finite positive *ε *< < 0.01, calculations reveal that *D *is positive but also much less than 0.01. These conditions guarantee that the second term in Equation 77 is positive for very small ε. In turn, because *D *increases from zero for any finite ε, its logarithm must also increase, and the third term is also therefore positive for small values of the scale factor. We note that since *t*_2 _decreases monotonically with *ε *(cf. Figure 5A), the first term in Equation 77 is negative. Despite this, numerical computation of the values of these three terms reveals that the latter two positive terms are larger in magnitude than the first for small ε, and dominate the behavior of $\frac{\partial t_{99}}{\partial \varepsilon }$ such that *t*_99 _initially increases, as shown in Figure 4C.

#### D. Systematic numerical sensitivity analysis of maximum capacity with PTC model

Because we did not have historical data with which to fit the maximum capacity with PTC model, we chose to perform the formal sensitivity analysis assuming that the pediatric rates were equal to those obtained from our fit for the adult center. We set the variance of each pediatric parameter to 35 percent of its value, and the adult parameter variances were set to the previously fitted values. We then performed the sensitivity analysis for the four outputs *t*_1,*a*_, *t*_1,*p*_, *t*_2 _and *t*_99 _as a function of the six parameters *k_paa_*, *k_pap_*, *k_pda_*, *k_pdp_*, *k_a_' *and *k_p_' *using the same procedure as described above. However, for the matrix **S**, all partial derivatives were evaluated numerically by incrementing each parameter by ± 0.001, and the average value for positive and negative increments was used for each matrix element *S_ij_*. The resulting variance matrix for the maximum capacity with PTC model is then

(78) $$V=\left(\begin{array}{cccccc} 0.016 & 0.200 & 0.095 & 0 & 0 & 0\\ 0.016 & 0.200 & 0.095 & 0.217 & 0 & 0\\ 0.078 & 0.996 & 0.003 & 0.002 & 0.022 & 4.645\\ 0.027 & 0.345 & 0.888 & 0.907 & 0.023 & 4.690 \end{array}\right)$$

where the first row gives the magnitudes of the effects upon *t*_1,*a *_of changing *k_paa_*, *k_pap_*, *k_pda_*, *k_pdp_*, *k_a_' *and *k_p_'*, the second the same values for *t*_1,*p*_, the third for *t*_2_, and the fourth for *t*_99_.

#### E. Pediatric disaster-related deaths are reduced by the availability of a PTC

**Figure 6:**
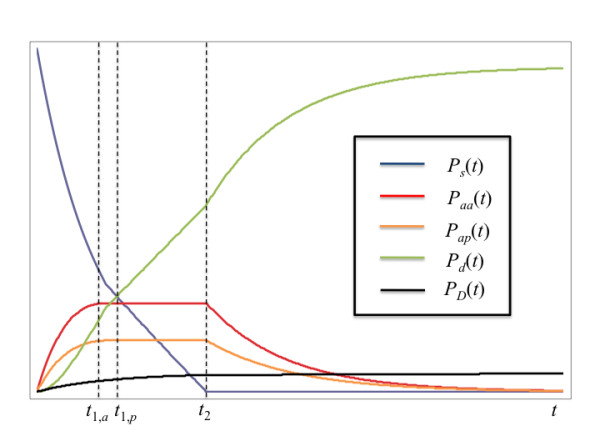
**Qualitative behavior of maximum capacity with PTC model with explicit death rates derived in Appendix B**. Blue curve: pediatric surge. Red curve: pediatric patients admitted to adult center. Orange curve: pediatric patients admitted to PTC. Green curve: living discharged patients. Black curve: deceased patients.

A severe limitation of both the maximum capacity and maximum capacity with PTC models is a lack of accounting for mortality. As a final modification to the maximum capacity with PTC model, we included explicit death rates for each of the populations: the surge, pediatric patients admitted to the adult or pediatric trauma centers, and patients after discharge. This model is fully developed in Appendix B, and its qualitative behavior is demonstrated in Figure 6. We chose to use as an outcome measure the proportion of patients deceased at *t *= 10 days, the time at which the field hospital in Reference 1 ceased operations. For this calculation, we began by assuming that after treatment and discharge, the death rate would equal the background age-adjusted death rate of the United States, which was approximately 8 per thousand per year in 2005 [43], or 2 × 10^-5 ^day^-1^. Although we could not find mortality data for admitted patients in the IDF field hospital described in Reference 1, we chose to use the figure of 8.6% mortality of admitted patients over 15 days, or 5.7 × 10^-3 ^day^-1^, from the Japanese experience after the 1995 Hanshin-Awaji earthquake [44]. Lastly, we based our estimate for the surge death rate, prior to admission and treatment, on data from the Chi-Chi earthquake in Taiwan in 1999, where it was reported that of all fatalities, 7% died while hospitalized [45]. We can therefore approximate the surge death rate by scaling our in-hospital rate from Reference 47 by 0.93/0.07, yielding a surge death rate of 0.076 day^-1^. We shall also assume that the death rate for patients admitted to the adult center is equal to that of those in the PTC. With these estimates, we find that with no PTC available, the proportion of the initial surge dead at *t *= 10 days is 24.0 percent, but with a PTC operating with the same admission and discharge rates as the adult center, this is decreased to 15.2 percent. This amounts to a reduction of the absolute mortality risk by 8.8 percent, and a relative mortality risk reduction of 37 percent, when a pediatric trauma center is available to admit and discharge patients at the same rates as those of the adult center.

## Discussion

### Summary of main results

A deterministic first-order population kinetics model has been presented to quantitatively describe the effect of the availability of a pediatric trauma center upon the time required to completely triage and definitively treat the pediatric cohort of a disaster surge. We first derived a simpler model to determine starting parameters from an historical example. We then proceeded to examine the effect of adding in the availability of a pediatric trauma center over a range of values for its efficiency as described by admission and discharge rates relative to the baseline values obtained for the adult center. While the time needed to triage or admit the entire pediatric surge cohort decreased with the availability of a PTC regardless of its efficiency, the time to discharge of the surge had a more complicated behavior: if *k_pdp_*is varied proportionally to the other parameters, the total discharge time *t*_99 _actually increases when the PTC is slow (with rates less than approximately 0.04 times those of the adult center), and only begins to fall below the baseline value of 10 days obtained from the historical example when the pediatric rate constants approach 0.4 times those of the adult center. If *k_pdp_*is set equal to *k_pda_*, however, the times needed for admission and discharge of the entire pediatric surge cohort are decreased from baseline regardless of how slow the admission or steady-state discharge rate from the PTC. Overall, if the PTC is able to admit and discharge patients at nearly the same rates as the adult center, the time needed to admit all pediatric patients is nearly halved (from 8 days to just over 4 days), and the time to complete discharge of the population is reduced by more than a third (from 10 days to a little more than 6.5 days). We note that in the setting of a disaster of sufficient scale to displace a significant enough proportion of the population, families may not be able to receive pediatric patients discharged to home, so the PTC discharge rate could be retarded by this effect, diminishing the predicted effect on the time to discharge of the entire surge. Despite this, the total admission time would always be decreased with PTC availability. Lastly, when death rates from previous disasters reported in the literature are incorporated into the maximum capacity with PTC model (cf. Appendix B), we find that the overall death rate would be decreased from 24.0% of the initial pediatric surge population to 15.2% when a PTC is available to admit and discharge pediatric patients at the same rates as the adult center, a relative mortality risk reduction of 37%.

The finding that *t*_99 _initially increases when the PTC rates are uniformly scaled can be described as a trapping effect. In other words, when the PTC becomes available to triage and admit pediatric disaster surge patients, if it cannot treat and discharge them fast enough, then the time needed for definitive disposition of the pediatric cohort is actually prolonged. This occurs because overall, when the PTC is much slower than the adult center, the population cohort admitted to the PTC stays there much longer on average than those patients admitted to the faster adult center. In the context of a real disaster, this would result in a prolonged use of specialized pediatric hospital resources, likely increased costs, and a decrease in the ability of the PTC to provide routine care to the non-surge pediatric population. We note, however, that regardless of how slowly the pediatric surge cohort can be discharged, the time needed to triage and admit the surge is always decreased in the setting of the availability of the PTC. We therefore speculate that the clinical result on the surge population would be minimal, but the impairment in ability of the PTC to provide routine care to the background population during this period of time would have to be considered in disaster and contingency planning.

Of equal interest to disaster planners are the results of the sensitivity analysis. We found that in the maximum capacity model (no PTC available), the discharge rate *k_d_*had the greatest influence on both the time to maximum load *t*_1 _and time to discharge of 99 percent of the surge population *t*_99 _(variance matrix elements, 0.184 and 0.815, respectively, Equation 72). In the setting where a PTC is available, the behavior of the total treatment time described in Figures 4B and 4D is consistent with this result, since the marked peaking of *t*_99 _shown in Figure 4B is completely abolished in Figure 4D when the pediatric center's pre-maximum load discharge rate *k_pdp_*is set equal to the fitted value of *k_d_*from the maximum capacity model in the sensitivity analysis. On the other hand, the effect of both the pre-maximum load admission rate *k_a_*as well as the steady-state discharge rate *k*' were found to contribute about equally to the variance of the time needed to admit the entire cohort *t*_2_. These results suggest that to maximize the efficiency of a given center to definitively treat a given surge cohort, the most important factor is rapid discharge of inpatients before the maximum surge capacity is reached. This observation is consistent with an analysis conducted in a large tertiary center undergoing relocation to a new facility, which found that expedited discharge of inpatients was an effective means of increasing hospital capacity over the short term [46]. On the other hand, if the most critical goal to planners is simply to triage and admit the surge, with less importance placed upon definitive treatment and discharge, the pre-maximum load admission rate and the steady-state discharge rate should be optimized.

For the maximum capacity with PTC model, the interpretation of the numerical sensitivity analysis is somewhat more complicated. For *t*_1,*a*_, the time to achieving maximum capacity of the faster adult center, Equation 78 suggests that the pediatric admission rate *k_pap_*makes the most important contribution (matrix element 0.200). This is because in the numerical sensitivity analysis, pediatric parameters were all varied by 35%, while the error in the fitted value of the adult rate *k_paa_*was set equal to the much smaller error in *k_a_*from the maximum capacity model. For the time to reach the maximum capacity of the slower pediatric trauma center, *t*_1,*p*_, the pediatric discharge rate *k_pdp_*dominates (matrix element 0.217), but the pediatric admission rate *k_pap_*contributes almost as much (matrix element 0.200). This result is not unreasonable given the explicit and implicit dependence of *t*_1*p *_upon both these rate constants (cf. Equation 43).

In contrast, *t*_2 _is strongly affected by the steady-state PTC discharge rate *k_p_*' (matrix element 4.645). The dependence of *t*_2 _on *k_p_*' can be explained by two factors: first, the fact that the relative error in this rate assumed for our sensitivity analysis (35%) is larger than that obtained for *k_a_*' in the fit, and second, due to the functional form of *t*_2_. Equation 48 shows that *t*_2 _is a linear function of *t*_1*p *_and *B*, and therefore depends implicitly on *k_pda_*and *k_pdp_*. It is inversely proportional to the sum of the adult and pediatric steady-state discharge rates, κ = *k_p_*' + *k_a_'*. We have observed that invariably, whenever *t*_1*p *_increases, regardless of whether *k_pda_*or *k_pdp_*is changed, *B *decreases. Therefore, the effect of changing either *k_pda_*or *k_pdp_*upon *t*_2 _is limited because of this antagonistic effect. In contrast, changing κ by varying *k_p_*' or *k_a_' *does not produce a compensatory change in either *t*_1*p *_or *B*, so the effect of *k_p_*' dominates.

Lastly, *t*_99 _depends most strongly on *k_p_'*, with the next strongest dependence on *k_pdp_*and *k_pda_*(matrix elements 4.690, 0.907, 0.888 respectively). Though we cannot write down an analytic expression for *t*_99 _in the maximum capacity with PTC model, we can make qualitative arguments based on the behavior of this parameter in the simpler maximum capacity model. Equation 51 reveals that in the simpler model, *t*_99 _depends explicitly on *t*_2 _and the discharge rate *k_d_*, with implicit dependence upon both *k_d_*and *k_a_*within the argument of the logarithm. It is reasonable to conclude that in the more complicated maximum capacity with PTC model, the dependence would be similar on *t*_2 _and the two discharge rate constants *k_pdp_*and *k_pda_*. Since we have seen that for the maximum capacity with PTC model, *t*_2 _is most sensitive to changes in *k_p_*', it follows by this reasoning that *k_p_*' will also have a large effect on *t*_99_. Moreover, we have already seen that in the simpler model, *k_d_*actually has the greatest effect on *t*_99_, so taken together, this combined with the qualitative argument discussed here provide a reasonable explanation for the sensitivity of *t*_99 _to *k_pdp_*and *k_pda_*in the maximum capacity with PTC model.

### Limitations of the model

The potential methodological weaknesses of the model must also be considered. First, as noted above, no distinction is made in the discharged populations of either the maximum capacity model, or the maximum capacity with PTC model, between patients discharged home or otherwise dispositioned, including discharge to nursing care centers, rehabilitation hospitals, and even death. Similarly, the death of patients in the surge population prior to triage and admission is not accounted for. This concern is addressed in full detail in Appendix B, where a more complicated version of the model including death rates is derived, and is implemented in additional files 1 and 2. We expect that a deterministic population kinetics approach will describe the behavior of the populations of interest only when they are sufficiently large. However, for very small populations the continuous mathematics used to derive our model would be expected to break down, and a discrete stochastic approach [47] might be more appropriate.

### Tradeoffs

An important consideration for disaster planners is the potential cost of various approaches to preparedness. Though our model provides a mathematical justification for the inclusion or use of a pediatric trauma center in the response to a disaster, it does not consider the monetary cost of establishing one, or the resources required to keep it in operation. The average cost of building a new hospital has been reported in the United States to be approximately 285 dollars per square foot as of 2003 [48], or 342 2011 dollars per square foot [49]. At our own facility, a new 460,000 square foot (42,700 m^2^) specialized children's hospital with 317 beds, a level I pediatric trauma center and supporting facilities cost 636 million dollars in 2011, a cost of nearly 1400 dollars per square foot [50]. Therefore, prior to committing to building such a facility, a careful accounting of the likelihood of various types of disaster occurring in the proposed construction area as well as the availability of rapid transportation to and capacities of already existing nearby centers would have to be performed. Alternatively, a different approach would be for planners at an established center to prepare mobile dedicated pediatric trauma center facilities similar to the mobile field hospital described in reference 1, available to be transported to the site of a disaster as needed. However, we speculate that this method, though much less expensive than building a new PTC, could possibly have detrimental effects on treatment of affected adult patients. For example, after prolonged operation, such facility would require resupply, and if a medical resupply shipment had to be parcelled out to the PTC in addition to competing adult centers in the affected area, the resulting relative shortage of resources in the adult centers might result in decreased rates of admission and discharge, and increased death rates, of adults. Such considerations, though beyond the scope of our model directly, would also have to be examined to allow for its use in disaster planning.

## Conclusions

The model presented here provides an analytical, closed-form description of the population dynamics of a disaster surge population treated either in the presence or the absence of a pediatric trauma center, is mathematically elementary and is simple to implement. Given that the proportion of children in the population is roughly twenty-five percent,^35 ^the potential influence of the availability of a specialized trauma center whose resources are devoted to the pediatric surge cohort must be taken into consideration by public health agencies. We have demonstrated how the model can be applied to an historical example to obtain starting parameters, and the hypothetical contribution of an available PTC can then be assessed as a function of how it compares in efficiency to the historical example. If detailed quantitative historical data that explicitly included a PTC as part of the response to a disaster became available, the model could be fit to these data and estimates of the model parameters could be obtained. While the costs of building and maintaining a PTC and the effects of its resource consumption on other hospitals must be taken into account, this deterministic kinetic model provides a new weapon in the armamentarium of disaster planners. Our approach can be used to provide a hypothetical estimate of how the response to an historical event could have been improved, as well as to extrapolate and predict potential responses to future events.

## Competing interests

The authors declare that they have no competing interests.

## Authors' contributions

ERB derived the numerical model, performed the calculations and constructed the additional files, and wrote the first draft of the manuscript. The model concept was devised by ERB, JRP, CJG, HRF, TCG and JSU. ERB, CJG, TCG and JSU assisted with critical revisions. All authors have read and approved the final manuscript.

## Appendix A

### General case of Eqs. 1-4 with surge delayed from inciting event

For all MCEs, there is a delay between the inciting event or exposure and the development of the associated patient surge. The approximation made in the treatment in this paper is that the delay is much smaller than any of the other timescales in the model (i.e., admission or discharge). This is an excellent approximation for sudden MCEs such as bombings, earthquakes or airplane crashes. However, for some classes of MCE, such as disease pandemics, radiation exposure events, floods, hurricanes, as well as the aftermath of more sudden types of insults considered above, the delay time between event and surge is of the same order of magnitude as these other timescales, and must be treated explicitly in the model.

We now present the general case only for Region I of the simpler model entailed by Eqs. 1-4, because the inclusion of the pediatric trauma center makes the equations significantly more complicated, with the introduction of an additional parameter (the delay time) and differential equations, with a limited contribution to any further physical or planning insight. The procedure for obtaining the solution in the maximum capacity and zero queue-length regimes (Regions II and III of the maximum capacity model), as well as the inclusion of a PTC, would be the same as that in the main text. In the general case of the model, in the absence of the pediatric trauma center, we would have four populations rather than the three in Eq. 1:

(A1) $$N_{e}\overset{k_{s}}{\underset{}{\to }}N_{s}\overset{k_{a}}{\underset{}{\to }}N_{a}\overset{k_{d}}{\underset{}{\to }}N_{d}$$

Here *k_s_*is the exposure or delay rate; the remaining rate constants are identical to those of Eq. 1. Also, instead of *N*_0 _instantaneous surge patients, we now have *N*_0 _exposed patients at time zero. The governing differential equations are then:

(A2) $$\frac{dN_{e}}{dt}=-k_{s}N_{e}$$

(A3) $$\frac{dN_{s}}{dt}=k_{s}N_{e}-k_{a}N_{s}$$

(A4) $$\frac{dN_{a}}{dt}=k_{a}N_{s}-k_{d}N_{a}$$

(A5) $$\frac{dN_{d}}{dt}=k_{d}N_{a}$$

The boundary conditions are:

(A6) $$N_{e}\left(t=0\right)=N_{0}$$

(A7) $$N_{s}\left(t=0\right)=N_{a}\left(t=0\right)=N_{d}\left(t=0\right)=0$$

(A8) $$N_{s}\left(t\to \infty \right)=N_{a}\left(t\to \infty \right)=N_{d}\left(t\to \infty \right)=0$$

The solution to (A2-A5) is then:

(A9) $$N_{e}\left(t\right)=N_{0}e^{-k_{s}t}$$

(A10) $$N_{s}\left(t\right)=N_{0}\frac{k_{s}}{k_{a}-k_{s}}\left(e^{-k_{s}t}-e^{-k_{a}t}\right)$$

(A11) $$\begin{array}{c} N_{a}\left(t\right)=N_{0}\frac{k_{a}k_{s}}{k_{a}-k_{s}}\left[\frac{1}{k_{d}-k_{s}}e^{-k_{s}t}\right)\\ \;\;\;\left(-\frac{1}{k_{d}-k_{a}}e^{-k_{a}t}-\left(\frac{1}{k_{d}-k_{s}}-\frac{1}{k_{d}-k_{a}}\right)e^{-k_{d}t}\right] \end{array}$$

(A12) $$\begin{array}{c} N_{d}\left(t\right)=N_{0}\frac{k_{s}k_{a}k_{d}}{k_{a}-k_{s}}\left[\frac{1}{k_{a}\left(k_{d}-k_{a}\right)}\left(e^{-k_{a}t}-1\right)\right)\\ \;\;\;-\frac{1}{k_{s}\left(k_{d}-k_{s}\right)}\left(e^{-k_{s}t}-1\right)\\ \left(\;\;\;+\frac{1}{k_{d}}\left(\frac{1}{k_{d}-k_{s}}-\frac{1}{k_{d}-k_{a}}\right)\left(e^{-k_{d}t}-1\right)\right] \end{array}$$

Similarly to the case of Eqs. 31 and 32, we can compute exactly the time of maximum expected surge by taking the derivative of A10 and setting it equal to zero, which gives:

(A13) tsmax=1ka-ks lnkaks

We note that in the context of disaster planning, A13 can either be used to predict the time of maximum surge, if estimates for *k_a_*and *k_s_*are known, or to constrain and relate *k_a_*to *k_s_*if the maximum surge time is known from historical or data or other predictive methods.

## Appendix B

### Maximum capacity with PTC model and explicit death rates

In this section we shall derive a version of the maximum capacity with PTC model where a background death rate of each population is included. For times at which neither the adult nor the pediatric center has reached maximum capacity (analogous to region I of Figure 3) the governing differential equations are:

(B1) $$\begin{array}{ll} 0\leq t<t_{1,a}:\; & \frac{dP_{s}}{dt}=-\left(k+\omega_{s}\right)P_{s}\;\\ \;\;\;\;\;\; & \frac{dP_{aa}}{dt}=k_{paa}P_{s}-\left(k_{pda}+\omega_{a}\right)P_{aa}\;\\ \;\;\;\;\;\; & \frac{dP_{ap}}{dt}=k_{pap}P_{s}-\left(k_{pdp}+\omega_{p}\right)P_{ap}\;\\ \;\;\;\;\;\; & \frac{dP_{d}}{dt}=k_{pda}P_{aa}+k_{pdp}P_{ap}-\omega_{d}P_{d}\;\\ \;\;\;\;\;\; & \frac{dP_{D}}{dt}=\omega_{s}P_{s}+\omega_{a}P_{aa}+\omega_{p}P_{ap}+\omega_{d}P_{d}\;\\ & \; \end{array}$$

where ω_s_, ω_a_, ω_p_, and ω_d _are the death rates for the surge, pediatric patients admitted to the adult center, pediatric patients admitted to the PTC, and discharged patients, respectively, *P_D_*(*t*) is the total number of deaths that have occurred at time *t*, and all other parameters are as defined in the main text. We introduce *J*, *K *and *L *as the boundary conditions for the deceased population *P_D_*(*t*) at *t_1, a_*, *t_1, p_*, and *t*_2_, respectively. We define

(B2) $$\begin{array}{c} \lambda_{s}=k+\omega_{s}\\ \lambda_{a}=k_{pda}+\omega_{a}\\ \lambda_{p}=k_{pdp}+\omega_{p} \end{array}$$

The solution to (B1) is:

(B3) $$\begin{array}{c} P_{s}\left(t\right)=e^{-\lambda_{s}t}\\ P_{aa}\left(t\right)=\frac{k_{paa}}{\lambda_{a}-\lambda_{s}}\left(e^{-\lambda_{s}t}-e^{-\lambda_{a}t}\right)\\ P_{ap}\left(t\right)=\frac{k_{pap}}{\lambda_{p}-\lambda_{s}}\left(e^{-\lambda_{s}t}-e^{-\lambda_{p}t}\right)\\ P_{d}\left(t\right)=\frac{1}{\omega_{d}-\lambda_{s}}\left(\frac{k_{pda}k_{paa}}{\lambda_{a}-\lambda_{s}}+\frac{k_{pdp}k_{pap}}{\lambda_{p}-\lambda_{s}}\right)\left(e^{-\lambda_{s}t}-e^{-\omega_{d}t}\right)\\ \;+\frac{k_{pda}k_{paa}}{\left(\omega_{d}-\lambda_{a}\right)\left(\lambda_{s}-\lambda_{a}\right)}\left(e^{-\lambda_{a}t}-e^{-\omega_{d}t}\right)\;\;\\ \;+\frac{k_{pdp}k_{pap}}{\left(\omega_{d}-\lambda_{p}\right)\left(\lambda_{s}-\lambda_{p}\right)}\left(e^{-\lambda_{p}t}-e^{-\omega_{d}t}\right)\\ P_{D}\left(t\right)=\frac{1}{\lambda_{s}}\left(1-e^{-\lambda_{s}t}\right)\left[\omega_{s}+\omega_{a}\frac{k_{paa}}{\lambda_{a}-\lambda_{s}}+\omega_{p}\frac{k_{pap}}{\lambda_{p}-\lambda_{s}}\right)\\ \;\;\left(+\omega_{d}\frac{1}{\omega_{d}-\lambda_{s}}\left(\frac{k_{pda}k_{paa}}{\lambda_{a}-\lambda_{s}}+\frac{k_{pdp}k_{pap}}{\lambda_{p}-\lambda_{s}}\right)\right]\\ \;+\frac{k_{paa}}{\lambda_{a}\left(\lambda_{s}-\lambda_{a}\right)}\left(1-e^{-\lambda_{a}t}\right)\left(\omega_{a}+\omega_{d}\frac{k_{pda}}{\omega_{d}-\lambda_{a}}\right)\\ \;+\frac{k_{pap}}{\lambda_{p}\left(\lambda_{s}-\lambda_{p}\right)}\left(1-e^{-\lambda_{p}t}\right)\left(\omega_{p}+\omega_{d}\frac{k_{pdp}}{\omega_{d}-\lambda_{p}}\right)\\ \;+\left(1-e^{-\omega_{d}t}\right)\left[\frac{k_{pda}k_{paa}}{\lambda_{s}-\lambda_{a}}\left(\frac{1}{\omega_{d}-\lambda_{s}}-\frac{1}{\omega_{d}-\lambda_{a}}\right)\right)\\ \left(\;\;+\frac{k_{pdp}k_{pap}}{\lambda_{s}-\lambda_{p}}\left(\frac{1}{\omega_{d}-\lambda_{s}}-\frac{1}{\omega_{d}-\lambda_{p}}\right)\right] \end{array}$$

The time at which the adult center's maximum capacity is reached is

(B4) t1,a=1λs-λa lnλsλa

For times at which the faster adult center has reached its maximum capacity, but the PTC has not (analogous to region II of Figure 3), the system's behavior is governed by

(B5) $$\begin{array}{ll} t_{1,a}\leq t<t_{1,p}:\; & \frac{dP_{s}}{dt}=-\lambda_{II}P_{s}-{\lambda_{a}}^{\prime }\;\\ \;\;\;\;\;\;\;\; & \frac{dP_{aa}}{dt}=0\;\\ \;\;\;\;\;\;\;\; & \frac{dP_{ap}}{dt}=k_{pap}P_{s}-\lambda_{p}P_{ap}\;\\ \;\;\;\;\;\;\;\; & \frac{dP_{d}}{dt}=k_{pdp}P_{ap}+{k_{pda}}^{\prime }-\omega_{d}P_{d}\;\\ \;\;\;\;\;\;\;\; & \frac{dP_{D}}{dt}=\omega_{s}P_{s}+{\omega_{a}}^{\prime }+\omega_{p}P_{ap}+\omega_{d}P_{d}\;\\ & \; \end{array}$$

where ω_a_' is the constant death rate of patients hospitalized in the adult center, and we have introduced

(B6) $$\begin{array}{c} \lambda_{II}=k_{pap}+\omega_{s}\\ {\lambda_{a}}^{\prime }={k_{a}}^{\prime }+{\omega_{a}}^{\prime } \end{array}$$

The solution to (B4) is

(B7) $$\begin{array}{c} P_{s}\left(t\right)=\alpha^{\prime }e^{-\lambda_{II}\left(t-t_{1,a}\right)}-\rho \\ P_{aa}\left(t\right)=C\\ P_{ap}\left(t\right)=\left(H+\varphi -\theta \right)e^{-\lambda_{p}\left(t-t_{1,a}\right)}\\ \;\;\;+\theta e^{-\lambda_{II}\left(t-t_{1,a}\right)}-\varphi \\ P_{d}\left(t\right)=\Gamma \left(e^{-\lambda_{p}\left(t-t_{1,a}\right)}-e^{-\omega_{d}\left(t-t_{1,a}\right)}\right)\\ \;\;\;+\Delta \left(e^{-\lambda_{II}\left(t-t_{1,a}\right)}-e^{-\omega_{d}\left(t-t_{1,a}\right)}\right)\\ \;\;\;+\Lambda \left(1-e^{-\omega_{d}\left(t-t_{1,a}\right)}\right)+Ee^{-\omega_{d}\left(t-t_{1,a}\right)}\\ P_{D}\left(t\right)=\left({\omega_{a}}^{\prime }+\Lambda \omega_{d}-\rho \omega_{s}-\varphi \omega_{p}\right)\left(t-t_{1,a}\right)\\ \;\;\;+\frac{1}{\lambda_{p}}\left(\left(H+\varphi -\theta \right)\omega_{p}+\Gamma \omega_{d}\right)\left(1-e^{-\lambda_{p}\left(t-t_{1,a}\right)}\right)\\ \;\;\;+\frac{1}{\lambda_{II}}\left(\alpha^{\prime }\omega_{s}+\theta \omega_{p}+\Delta \omega_{d}\right)\left(1-e^{-\lambda_{II}\left(t-t_{1,a}\right)}\right)\\ \;\;\;+\left(\Lambda +\Delta +\Gamma -E\right)\left(e^{-\omega_{d}\left(t-t_{1,a}\right)}-1\right)+J \end{array}$$

Where

(B8) $$\begin{array}{c} A=e^{-\lambda_{s}t_{1,a}}\\ C=\frac{k_{paa}}{\lambda_{a}-\lambda_{s}}\left(e^{-\lambda_{s}t_{1,a}}-e^{-\lambda_{a}t_{1,a}}\right)\\ H=\frac{k_{pap}}{\lambda_{p}-\lambda_{s}}\left(e^{-\lambda_{s}t_{1,a}}-e^{-\lambda_{p}t_{1,a}}\right)\\ E=\frac{1}{\omega_{d}-\lambda_{s}}\left(\frac{k_{pda}k_{paa}}{\lambda_{a}-\lambda_{s}}+\frac{k_{pdp}k_{pap}}{\lambda_{p}-\lambda_{s}}\right)\left(e^{-\lambda_{s}t_{1,a}}-e^{-\omega_{d}t_{1,a}}\right)\\ \;+\frac{k_{pda}k_{paa}}{\left(\omega_{d}-\lambda_{a}\right)\left(\lambda_{s}-\lambda_{a}\right)}\left(e^{-\lambda_{a}t_{1,a}}-e^{-\omega_{d}t_{1,a}}\right)\\ \;+\frac{k_{pdp}k_{pap}}{\left(\omega_{d}-\lambda_{p}\right)\left(\lambda_{s}-\lambda_{p}\right)}\left(e^{-\lambda_{p}t_{1,a}}-e^{-\omega_{d}t_{1,a}}\right)\\ J=\frac{1}{\lambda_{s}}\left(1-A\right)\left[\omega_{s}+\omega_{a}\frac{k_{paa}}{\lambda_{a}-\lambda_{s}}+\omega_{p}\frac{k_{pap}}{\lambda_{p}-\lambda_{s}}\right)\\ \;\;\;\left(+\omega_{d}\frac{1}{\omega_{d}-\lambda_{s}}\left(\frac{k_{pda}k_{paa}}{\lambda_{a}-\lambda_{s}}+\frac{k_{pdp}k_{pap}}{\lambda_{p}-\lambda_{s}}\right)\right]\\ \;+\frac{k_{paa}}{\lambda_{a}\left(\lambda_{s}-\lambda_{a}\right)}\left(\omega_{a}+\omega_{d}\frac{k_{pda}}{\omega_{d}-\lambda_{a}}\right)\left(1-e^{-\lambda_{a}t_{1,a}}\right)\\ \;+\frac{k_{pap}}{\lambda_{p}\left(\lambda_{s}-\lambda_{p}\right)}\left(\omega_{p}+\omega_{d}\frac{k_{pdp}}{\omega_{d}-\lambda_{p}}\right)\left(1-e^{-\lambda_{p}t_{1,a}}\right)\\ \;+\left(1-e^{-\omega_{d}t_{1,a}}\right)\left[\frac{k_{pda}k_{paa}}{\lambda_{s}-\lambda_{a}}\left(\frac{1}{\omega_{d}-\lambda_{s}}-\frac{1}{\omega_{d}-\lambda_{a}}\right)\right)\\ \;\;\;\left(+\frac{k_{pdp}k_{pap}}{\lambda_{s}-\lambda_{p}}\left(\frac{1}{\omega_{d}-\lambda_{s}}-\frac{1}{\omega_{d}-\lambda_{p}}\right)\right] \end{array}$$

and

(B9) $$\begin{array}{c} \alpha^{\prime }=A+\rho \\ \rho =\frac{{\lambda_{a}}^{\prime }}{\lambda_{II}}\\ \theta =\frac{\alpha^{\prime }k_{pap}}{\lambda_{p}-\lambda_{II}}\\ \varphi =\frac{\rho k_{pap}}{\lambda_{p}}\\ \Gamma =\frac{\left(H+\varphi -\theta \right)k_{pdp}}{\omega_{d}-\lambda_{p}}\\ \Delta =\frac{\theta k_{pdp}}{\omega_{d}-\lambda_{II}}\\ \Lambda =\frac{{k_{a}}^{\prime }-\varphi k_{pdp}}{\omega_{d}} \end{array}$$

We note that we have introduced a new boundary condition *J *for the fraction of patients who are deceased at *t_1, a_*. The time at which the pediatric center reaches its maximum capacity is again obtained by maximizing *P_ap_*(*t*) in B6 and is

(B10) $$\begin{array}{c} t_{1,p}=t_{1,a}\\ \;+\frac{1}{\lambda_{p}-\lambda_{II}}ln\left[\frac{\lambda_{p}}{\lambda_{II}}+\frac{\left(\lambda_{II}-\lambda_{p}\right)}{\alpha^{\prime }k_{pap}\lambda_{II}}\left(\frac{{\lambda_{a}}^{\prime }k_{pap}}{\lambda_{II}}+H\lambda_{p}\right)\right] \end{array}$$

After this time, and until the surge is exhausted, both the adult and pediatric centers are at steady state and can only admit a patient if another is discharged or dies:

(B11) $$\begin{array}{ll} t_{1,p}\leq t<t_{2}:\; & \frac{dP_{s}}{dt}=-\mu -\omega_{s}P_{s}\;\\ \;\;\;\;\;\;\; & \frac{dP_{aa}}{dt}=\frac{dP_{ap}}{dt}=0\;\\ \;\;\;\;\;\;\; & \frac{dP_{d}}{dt}=\kappa -\omega_{d}P_{d}\;\\ \;\;\;\;\;\;\; & \frac{dP_{D}}{dt}=\omega_{s}P_{s}+\omega_{d}P_{d}+{\omega_{a}}^{\prime }+{\omega_{p}}^{\prime }\;\\ & \; \end{array}$$

where we have introduced

(B12) $$\mu =\kappa +{\omega_{a}}^{\prime }+{\omega_{p}}^{\prime }$$

and ω_p_' is the constant death rate of patients hospitalized in the pediatric center during this time period. The solution to B9 is

(B13) $$\begin{array}{c} P_{s}\left(t\right)=-\frac{\mu }{\omega_{s}}+\left(B+\frac{\mu }{\omega_{s}}\right)e^{-\omega_{s}\left(t-t_{1,p}\right)}\\ P_{aa}\left(t\right)=C\\ P_{ap}\left(t\right)=D\\ P_{d}\left(t\right)=\frac{\kappa }{\omega_{d}}+\left(F-\frac{\kappa }{\omega_{d}}\right)e^{-\omega_{d}\left(t-t_{1,p}\right)}\\ P_{D}\left(t\right)=K+\left(F-\frac{\kappa }{\omega_{d}}\right)\left(1-e^{-\omega_{d}\left(t-t_{1,p}\right)}\right)\\ \;\;\;+\left(B+\frac{\mu }{\omega_{s}}\right)\left(1-e^{-\omega_{s}\left(t-t_{1,p}\right)}\right) \end{array}$$

where *K *is the fraction of deceased patients at *t_1, p_*and the constants are given by

(B14) $$\begin{array}{c} B=\alpha^{\prime }e^{-\lambda_{II}\left(t_{1,p}-t_{1,a}\right)}-\rho \\ D=\left(H+\varphi -\theta \right)e^{-\lambda_{p}\left(t_{1,p}-t_{1,a}\right)}\\ \;\;+\theta e^{-\lambda_{II}\left(t_{1,p}-t_{1,a}\right)}-\varphi \\ F=\Gamma \left(e^{-\lambda_{p}\left(t_{1,p}-t_{1,a}\right)}-e^{-\omega_{d}\left(t_{1,p}-t_{1,a}\right)}\right)\\ \;+\Delta \left(e^{-\lambda_{II}\left(t_{1,p}-t_{1,a}\right)}-e^{-\omega_{d}\left(t_{1,p}-t_{1,a}\right)}\right)\\ \;+\Lambda \left(1-e^{-\omega_{d}\left(t_{1,p}-t_{1,a}\right)}\right)+Ee^{-\omega_{d}\left(t_{1,p}-t_{1,a}\right)}\\ K=\left({\omega_{a}}^{\prime }+\Lambda \omega_{d}-\rho \omega_{s}-\varphi \omega_{p}\right)\left(t_{1,p}-t_{1,a}\right)\\ \;+\frac{1}{\lambda_{p}}\left(\left(H+\varphi -\theta \right)\omega_{p}+\Gamma \omega_{d}\right)\left(1-e^{-\lambda_{p}\left(t_{1,p}-t_{1,a}\right)}\right)\\ \;+\frac{1}{\lambda_{II}}\left(\alpha^{\prime }\omega_{s}+\theta \omega_{p}+\Delta \omega_{d}\right)\left(1-e^{-\lambda_{II}\left(t_{1,p}-t_{1,a}\right)}\right)\\ \;+\left(\Lambda +\Delta +\Gamma -E\right)\left(e^{-\omega_{d}\left(t_{1,p}-t_{1,a}\right)}-1\right)+J \end{array}$$

The time *t*_2 _at which the surge is exhausted is determined by setting the first expression in B11 equal to zero and is

(B15) t2=t1,p+1ωs ln1+Bωsμ

For times after *t*_2 _the adult and pediatric centers resume discharges at pre-maximum capacity rates:

(B16) $$\begin{array}{ll} t>t_{2}:\; & \frac{dP_{s}}{dt}=P_{s}\left(t\right)=0\;\\ \;\;\;\; & \frac{dP_{aa}}{dt}=-\lambda_{a}P_{aa}\;\\ \;\;\;\; & \frac{dP_{ap}}{dt}=-\lambda_{p}P_{ap}\;\\ \;\;\;\; & \frac{dP_{d}}{dt}=k_{pda}P_{aa}+k_{pdp}P_{ap}-\omega_{d}P_{d}\;\\ \;\;\;\; & \frac{dP_{D}}{dt}=\omega_{aa}P_{aa}+\omega_{p}P_{ap}+\omega_{d}P_{d}\;\\ & \; \end{array}$$

The solution to B13 is

(B17) $$\begin{array}{c} P_{aa}\left(t\right)=Ce^{-\lambda_{a}\left(t-t_{2}\right)}\\ P_{ap}\left(t\right)=De^{-\lambda_{p}\left(t-t_{2}\right)}\\ P_{d}\left(t\right)=C\frac{k_{pda}}{\omega_{d}-\lambda_{a}}\left(e^{-\lambda_{a}\left(t-t_{2}\right)}-e^{-\omega_{d}\left(t-t_{2}\right)}\right)\\ \;\;\;+D\frac{k_{pdp}}{\omega_{d}-\lambda_{p}}\left(e^{-\lambda_{p}\left(t-t_{2}\right)}-e^{-\omega_{d}\left(t-t_{2}\right)}\right)\\ \;\;\;+Ge^{-\omega_{d}\left(t-t_{2}\right)}\\ P_{D}\left(t\right)=L+\frac{D}{\lambda_{p}}\left(\omega_{p}+\frac{\omega_{d}k_{pdp}}{\omega_{d}-\lambda_{p}}\right)\left(1-e^{-\lambda_{p}\left(t-t_{2}\right)}\right)\\ \;\;\;+\frac{C}{\lambda_{a}}\left(\omega_{a}+\frac{\omega_{d}k_{pda}}{\omega_{d}-\lambda_{a}}\right)\left(1-e^{-\lambda_{a}\left(t-t_{2}\right)}\right)\\ \;\;\;+\left(G-C\frac{k_{pda}}{\omega_{d}-\lambda_{a}}-D\frac{k_{pdp}}{\omega_{d}-\lambda_{p}}\right)\left(1-e^{-\omega_{d}\left(t-t_{2}\right)}\right) \end{array}$$

where *L *is the fraction of deceased patients at *t*_2 _and the constants not yet defined are given by

(B18) $$\begin{array}{c} G=\frac{\kappa }{\omega_{d}}+\left(F-\frac{\kappa }{\omega_{d}}\right)e^{-\omega_{d}\left(t_{2}-t_{1,p}\right)}\\ L=K+\left(F-\frac{\kappa }{\omega_{d}}\right)\left(1-e^{-\omega_{d}\left(t_{2}-t_{1,p}\right)}\right)\\ \;\;\;+\left(B+\frac{\mu }{\omega_{s}}\right)\left(1-e^{-\omega_{s}\left(t_{2}-t_{1,p}\right)}\right) \end{array}$$

## Supplementary Material

Additional file 1 **We shall briefly describe here the first Microsoft Excel spreadsheet that accompanies this paper**. The spreadsheet, titled *MC and MCPTC models.xls *[Additional File 1], allows the reader to enter values for the rates of admission and discharge, both for the maximum capacity model (worksheet tab "Max capacity model") and for the maximum capacity with pediatric trauma center model (worksheet tab "Max capacity model with PTC"). The behavior of each population and resulting timescales in the two models, as well as the boundary conditions described in the text and illustrated in Figure 3 for the latter model, are calculated and displayed in the labelled boxes as described by header notes on each worksheet.
Regarding the calculations themselves, the usual Excel worksheet functions EXP and LN are employed, but in order to properly calculate the behavior of the populations in each regime, conditional logic statements must be constructed. In computer programming languages, statements of the form if...then; else... must be coded in such cases. In the case of the maximum capacity model, for example, this involves evaluating different expressions for each population for the time regimes *t *<*t*_1_, *t*_1 _≤ *t *<*t*_2_, and *t *≥ *t*_2_. To accomplish this in Excel, the worksheet function IF(*logical test*, *expression 1*, *expression 2*) can be constructed to contain nested conditions [51]. In other words, since we require a statement of the form
if *t *<*t*_1 _then *f*_1_(*t*);
else if *t*_1 _≤ *t *<*t*_2 _then *f*_2_(*t*);
else *f*_3_(*t*);
end if
where *f*_1_(*t*), etc. are the functions that describe the desired behavior in each region, the corresponding Excel statement is
IF("*t *<*t*_1_", "*f*_1_(*t*)", IF(AND("*t *≥ *t*_1_", "*t *<*t*_2_"), "*f*_2_(*t*)", "*f*_3_(*t*)")
where the quotation marks reflect the fact that the enclosed statements are just shorthand for demonstration purposes, and in an Excel worksheet must be properly formatted Excel statements and functions of worksheet cells. For a demonstration please see the additional files.
We also reiterate here that the derivation for the maximum capacity with PTC model (section **III **of **Methods **in the manuscript) assumes that the pediatric trauma center is, at most, no faster than the adult center. This constrains the values that can be input by the user for the discharge rate of pediatric patients from the PTC to be less than or equal to that of their discharge from the adult center, i.e. *k_pdp _*≤ *k_pda_*. If the user sets *k_pdp_*>*k_pda_*, the admitted population of the pediatric center reaches its maximum capacity before the adult center, and the derivation (which assumed the opposite is true) is invalid. To explore the behavior of the model when the PTC is faster (not addressed in this paper), the user could set the *pediatric *rate constants to the desired or known baseline adult center values, and then vary the *adult *rates to be as rapid as desired. This amounts to simply relabeling all the rate constants for the PTC as those for the adult center and vice versa. Click here for file

Additional file 2 **The second Excel spreadsheet [Additional File **2] **incorporates both the maximum capacity with PTC model and explicit death rates for each pediatric population as derived in Appendix B**. The user interface is similar to that of the first file, with modifiable inputs in bold and boxes color-coded for inputs (blue), dimensionless constants (green), and outputs and boundary conditions (orange). The lower right hand corner of the orange box also displays the proportions of discharged and deceased patients. Because of the greater complexity of this version of the model, we also included worksheet tabs for regions I, II, III, and IV separately for demonstration purposes, which can be found to the right of the main model tabs labelled "MCPTC with deaths, PTC unavail" and "MCPTC with deaths." Click here for file
